# Book Reviews

**Published:** 1912-11

**Authors:** 


### BOOK REVIEWS

Making Good on Private Duty.—Practical Hints to Graduate Nurses, by Harriet Camp Lounsbery, R. N., Charleston, W. Va., published by J. B. Lippincott Co., Philadelphia, 208 pages, $1.00.

While the book contains many little hints as to diet, details of nursing, etc., it deals mainly with the relation of nurse to patient and physician. If every nurse read and followed its teachings, we would meet fewer objections to the employment of a trained nurse on the ground not of direct economy, but that "she is so much trouble."

Catalogue of Medical and Surgical Works Published in the U. S., alphabetically arranged by authors and classified under subjects, 1912-13. W. B. Saunders Co., Philadelphia.

This is a most valuable list of books in print (not necessarily published in the years mentioned). The only favors shown their own house by the publishers is the inclusion of a few advertisements of their own books and the use of the full name instead of an abbreviation to indicate the publishing house. We would suggest, however, a more careful discrimination of authors of the same surname. The editor, for instance has no desire to claim the authorship of "Elementary Organic Analysis." He is surprised to learn that a little book on "Practical Dietetics" is still in print under a slightly different title. Golden Rules of Dietetics is not published by "Mo.," (John P. Morton & Co., of Louisville,) but by "Mb.," The C. V. Mosby Co., of St. Louis. We would recommend a systematic study of this catalogue by prospective buyers and would remind our readers that they can usually be supplied through the BUFFALO MEDICAL JOURNAL.

---

THE WASSERMANN REACTION.—Its Technic and Practical Application in the Diagnosis of Syphilis, by John W. Marchildon, B. S., M. D., St. Louis, published by the C. V. Mosby Co., St. Louis. 103 pages, 11 illustrations and colored frontispiece. $1.50.

This book, like practically all emanating from this house, depends, not upon the author but upon what he knows, not upon the possibility of competing in already well supplied markets by offering staple goods bearing a name that it is hoped will lure purchasers from other stalls but upon the delivery of goods not elsewhere on sale. The matter is concisely and definitely presented. After a detailed consideration of the evidence regarding the appearance of the reaction in other diseases, the author concludes that, properly conducted, it is specific for syphilis. In over 700 cases of syphilis treated by mercury, the author found that the reaction disappeared in over 75 per cent. and was uninfluenced in none.

---

THE BLOOD OF THE FATHERS.—A play in four acts by G. Frank Lydstorm, Chicago, published by the Riverton Press, 245 pages, $1.25.

This is not strictly a medical work, although it deals with the typic high-pressure surgeon (a type mainly of lay creation) and ascribes the significant incidents in the plot to heredity. It is an exciting story of the conflict between professional and domestic interests, culminating with kleptomania and hydrocyanic acid on the part of the doctor's wife, thus leaving him free for a sequel. In fiction, undesirable persons have a tendency to remove themselves at critical periods, to the satisfaction, tinged with forgiveness and regret, of all concerned. In real life, similarly undesirable persons seem singularly endowed with resistance to shock and infection, suicidal impulse, etc., and to attain a considerable degree of longevity.

---

THE PRACTICE OF MEDICINE.—A manual for Students and Practitioners. By Hughes Dayton, M. D., formerly of the Cornell University Medical School, New York. New (2d) edition, thoroughly revised. 12mo, 326 pages. Cloth $1.00, net. The Medical Epitome Series. Lea & Febiger, publishers, Philadelphia and New York, 1912.

While it is the custom to condemn quiz compends and condensed medical works of similar nature, they will persist just so long as the examination system requires attainments best or at least most easily secured through their use. Personally, we do not share this prejudice. The late Henry F. Formad, in teaching pathology used to lay stress on the idea that the man who simply knew what diseases might involve a given organ or might give rise to a group of symptoms, was, on the whole, more apt to make a correct diagnosis than the one much more skilled in pathologic and diagnostic methods but with a hazy notion of the conditions that might be encountered. Quite apart from the needs of the under-graduate student, the practitioner is likely to develop skill and information in detail but to lose his bird's-eye view of diseases. Hence the value of works like the present, which enable one to refresh his memory all the better by omitting a mass of detail already mastered or easily acquired. The trend of modern thought is well illustrated by the fact that this excellent compend commences with a discussion of immunity and ignores temperament and diathesis, the tongue, pulse, etc., with which all orthodox courses in medicine formerly started.

---

Elementary Bacteriological and Protozoology: the Microbiological Causes of the Infectious Diseases. By Herbert Fox, M. D., Director of the William Pepper Laboratory of Clinical Medicine in the University of Pennsylvania. 12mo., 237 pages, with 67 engravings and 5 colored plates. Cloth, $1.75, net. Lea & Febiger, Philadelphia and New York, 1912.

One of the best tests of a man's attainments is what he does not know. A few years ago, we had most elaborate schemes of the life cycle of the variola-vaccinia organism. Fox briefly discusses small pox as a disease of unknown origin. Ultramicroscopic bacteria are not even indexed. Throughout, the text is concise and accurate and the book may be characterized as anaerothermic.

---

Materia Medica and Therapeutics. By Reynold Webb Wilcox, M. A., M. D., LL. D., New York. Published by P. Blakiston's Son & Co., Philadelphia, 8th revised edition, cloth, 832 pages, $3.00.

Part I deals with definitions, pharmacopoeial preparations, prescribing, etc., and then discusses the materia medica under the general divisions of Inorganic including non-metals and metals; and Organic, including synthetics, vegetable and animal drugs, the last two being sub-classified according to chief pharmacologic actions. Part II discusses pharmacology and therapeutics. The older technical terms indicating physiologic action, are retained only in subheadings. For example, there is a division dealing with "drugs acting upon organisms which infect the human body, or upon processes going on within it;" "drugs acting on the blood" are subdivided according to their action on the plasma, red corpuscles, white corpuscles and coagulability. The author, by avoiding prolix statements, has condensed a complete work into reasonable limits and the publishers have struck the happy medium between fine type and lack of paragraphing, and unnecessary spreading of printed matter. They have also used a paper which is thin but not transparent nor friable. We mention these details to show how a full text book on a lengthy subject has been put into a comparatively small, light and in either sense, a handy volume.

---

**Text Book of Human Physiology, by Albert P. Brubaker, A. M., M. D., Philadelphia, published by P. Blakiston's Son & Co., Philadelphia, 4th edition, 736 pages, 1 colored plate and 377 illustrations, $3.00.**

This edition is dedicated to "Kenneth M. Blakiston, Loyal Friend, Courteous Gentleman, Generous Publisher," a tribute not only well deserved personally, but a wholesome precedent for the recognition by the medical profession generally of the various professions which assist in various ways in the advance of medical knowledge. The interminable chapter on muscular contractility, so often found in text books on physiology, is condensed within reasonable limits, and the various topics are well treated in the light of modern discoveries. We notice two minor flaws: the special senses are not clearly classified and listed and the section on articulation (speech) is quite inadequate. For instance, it is stated that the vowel sounds, a, e, i, o, u, are laryngeal sounds modified by the superposition and reinforcement of certain over tones developed in the mouth and pharynx. In reality, the laryngeal sound has nothing to do with the vowel sound, as the latter are clearly differentiated in whispering when the larynx is not in use, and even after the removal of the larynx altogether, as for cancer. Again, the vowel sounds altogether number about 18, some of which are and some of which are not found in English and none of which is clearly indicated by letters in English, without arbitrary diacritic marks. Konig is quoted as ascribing certain vibratory values to the different vowel sounds but it should be stated whether the letters a, e, i, o, u, represent the German sounds or not. I, as pronounced in English certainly does not correspond to 3760 vibrations or any other single number, since it is a diphthong. The consonants likewise are very inadequately discussed.

---

**A Practical Text Book of the Diseases of Women, by Arthur N. H. Lewers, M. D., F. R. C. P., London, published by Paul B. Hoeber, New York. 540 pages, 258 illustrations in the text and 18 plates. 7th edition, $4.00.**

Hoeber's books are of the highest standard of print and paper and he has a reputation for securing authors of practical ability. There is not much opportunity for divergence in a text book on a standard subject and no attempt has been made at an artificial appearance of originality by sacrificing the natural arrangement. We can say little, therefore except in general terms of commendation.

---

PHARMACOLOGY, by Maurice Vejux Tyrode, M. D., published by P. Blakiston's Son & Co., Philadelphia, 2d edition, 288 pages, $1.50.

After a general consideration of definitions and principles, the author divides drugs into those whose constitutional action is in greater prominence, ferments, secretions and extracts of animal organs, drugs whose local action is more in evidence and drugs of inorganic origin. Groups are named after typic drugs, as "Group of amyl nitrite," so far as possible. In the present state of science, this plan impresses us as most sensible. The book was evidently prepared for use by his classes at Harvard but deserves attention by the graduate as well.

---

FURTHER RESEARCHES INTO INDUCED CELL REPRODUCTION AND CANCER, by H. C. Ross, J. W. Cropper and E. H. Ross. Published by John Murray, London, as part of the McFadden Researches. 125 pages, illustrated in color, $1.00 (Vol. II.)

As was the case with Volume I, these papers deal with controversial problems and, while of inestimable value to the special student, lack the completeness, systematic arrangement and, let us say, dogmatism, demanded by the ordinary reader.

---

N. Y. STATE MUSEUM BULLETIN No. 158 (April 1, 1912),

This issue, while theoretically the fortnightly bulletin, is an elaborate report of the Director, Prof. John M. Clarke, and of his co-workers in the fields of archaeology, geology, etc. The salvage and loss in the burning of the State Capitol, are reported in detail and, fortunately, illustrations and descriptions of certain lost specimens, are extant.

BUREAU OF AMERICAN ETHNOLOGY, Bulletin No. 52, Early Man in South America, By Ales Hrdlicka, Curator of the Division of Physical Anthropology, U. S. National Museum, in collaboration with W. H. Holmes, Bailey Willis, Fred. Eugene Wright and Clarence N. Fenner.

This Bulletin includes a thorough study of various discoveries by many explorers, and of a personal exploration in 1910, the principal issue being the antiquity of man in the New World. Without entering into details nor attempting to review the multitude of highly interesting scientific observations made along many lines, the general conclusion may be stated that in South America, as in North America, no indisputable nor even probable evidence exists of anything approaching geologic nor archaeologic antiquity. In other words, the expression "New World" is scientifically accurate so far as the Human race is concerned.

---

A Treatise on Diseases of the Hair, by George Thomas Jackson, M. D., Professor of Dermatology in the College of Physicians and Surgeons, Medical Department of Columbia University, and Charles Wood McMurtry, M. D., Instructor in Dermatology in the College of Physicians and Surgeons, Medical Department of Columbia University, New York. Octavo, 366 pages, with 109 engravings and 10 colored plates. Cloth, $3.75, net. Lea & Febiger, Philadelphia and New York, 1912.

There is no better illustration of the development of a true scientific spirit in the profession than a work of this sort, dealing systematically with structures formerly considered "infra dig." And, when we consider the mortification and, especially among women, the really serious social and economic handicap entailed by loss or deterioration of the hair, it is quite proper to include the practical clinical and therapeutic portions of the book with the humanitarian value of treatises dealing with other parts of the body. A preliminary section deals with the anatomy (including histology) and physiology, (including hygiene) of the hair and related dermal organs. We would suggest that the discussion of types of hair might well be extended so as to include racial differences.

---

A Manual of Chemistry.—A guide to Lectures and Laboratory Work for Beginners in Chemistry. A textbook specially adapted for students of medicine, pharmacy and dentistry, by W. Simon, Ph. D., M. D., Professor of Chemistry in the College of Physicians and Surgeons, Baltimore, and in the Baltimore College of Dental Surgery; Emeritus Professor in the Maryland College of Pharmacy; and Daniel Base, Ph. D., Professor of Chemistry in the University of Maryland. New (10th) edition, enlarged and thoroughly revised. Octavo, 774 pages, with 82 engravings and 9 colored plates, illustrating 64 of the most important chemical tests. Cloth, $3.00, net. Lea & Febiger, Philadelphia and New York, 1912.

The work is divided into sections on Chemical Physics, Principles of Chemistry, Non-Metals, Metals, Analytical Chemistry, Organic Chemistry, Physiological Chemistry. It is further divided into sections like short chapters. The depth to which the various topics are probed, is well adapted to the requirements of the medical student and physician but tables for reference, tabular groupings and typographic indications of relations of subdivisions to larger units of thought, so useful in aiding the beginner to grasp a subject, are not employed to the extent desirable and now common. For instance, we find no complete list of atomic weights, valence, specific gravity, specific heat, and the only full list of elements is divided into three groups according to their "great and general interest," "special use made of them," and their lack of anything but scientific interest, without any reference to their properties or relations. The modern tendency to revive, in modified form, the ancient conception of transmutability is ignored, as is the very plausible theory of the origin of natural gas independent of plant and animal remains. It would be the height of injustice to leave the impression that the book is unsystematic. The style is lucid, even entertaining and general principles are thoroughly taught in spite of the lack of detail so desirable for reference in tables. If edited by one with a genius for arrangement to afford bird's-eye views, the book would be ideal.

---

THE PRACTITIONER'S ENCLYCLOPEDIA OF MEDICINE AND SURGERY, in all their branches. Edited by J. Keogh Murphy, M. C., F. R. C. S., London, published by the Oxford University Press. 1423 pages, illustrated, cloth, $7.00, half leather, $8.00.

This is a truly elegant volume, systematically arranged under general divisions of Medicine, Surgery and various special branches. The alphabetic index facilitates reference. Of course, no single volume can literally fulfill the implication of the title. Foster's Encyclopaedia, for example, over 20 years ago, devoted 5 volumes to the endeavor, each of them with more pages and in much finer print than this. But the abandonment of the alphabetic arrangement of separate articles, each disappointingly brief and incomplete, has made possible a presentation of practical medical knowledge, mainly of a clinical nature, in an orderly way and to an extent that would seem impossible. Good judgment has been shown in gauging the discussion of laboratory tests, etc., to the equipment of the practitioner. Moreover, as there never can be an absolutely complete and stable encyclopaedia of a rapidly progressing science, it is wise to put reasonable limits to the scope and the cost of any work of this nature. In some respects, for example the treatment of medico-legal problems, the requirements of the non-British reader are not and could not have been fulfilled. Such statements as that the amount of sugar in diabetes may reach 12,000 grains would also be more intelligible to the American and, a fortiori, to the continental European reader, if expressed in metric units.

---

THE PRINCIPLES OF HUMAN PHYSIOLOGY, by Ernest Henry Starling, M. D., (London), F. R. C. P., F. R. S., Lodrell, Professor of Physiology in University College, London. Octavo, 1423 pages, with 564 illustrations, some in color. Cloth, $5.00, net. Lea & Febiger, Philadelphia and New York, 1912.

This is the kind of book in which one finds not only the expected but the unexpected. It might be called a quantitative physiology, in contrast to the ordinary qualitative text book. The chemistry of the body is carried back to the lightning flash which combines atmospheric nitrogen into ammonium nitrite and the synthesis of nitrogen is carried through the action of symbiotic bacteria of vegetable roots into animal chemistry. When a subject is mentioned, as ferments, calories, proteids, etc., complete lists are given. The work is quantitative not only in presenting numbers, as of percentage composition, amounts of excretory principles amounts of substances digested, etc., but in the free use of algebrais formulae, and, under the discussion of the eye, of trigonometry. Through the index, the reader can find much information for which he has been seeking in vain and we confess personally to have come upon several delightfully interesting topics of whose very existence we were ignorant. Yet the book is by no means difficult reading; its very thoroughness brings together blind passages and one subject is elucidated by being brought into relation with another. We would not give the impression that the work consists merely of statistics of physiologic chemistry and physics. It is a genuine physiology, incorporating much recent discovery regarding immunization, the ductless glands, gastric, faecal, urinary blood and other objects of laboratory investigation whose relation physiology we have scarcely appreciated.

---

Pharmacology and Therapeutics, H. C. Wood, Jr., M. D., Philadelphia, published by the J. B. Lippincott Co., Philadelphia, 429 pages, $4.00.

Dr. Wood is both fortunate and unfortunate in the fact that his family has been so active in the study of drugs for nearly a century. An inspection of his book convinces one that while he may properly benefit by the association of Wood with drugs, in the professional mind, he has put into it a proper degree of original investigation and study. The work itself follows the customary pharmacologic arrangement, is systematic and thorough.

---

Pathology and Treatment of Diseases of Women, 4th edition, by A. Martin and Ph. Jung of Greifswald, Germany, only authorized English translation by Henry Scmitz, M. D., Chicago. Published by the Rebman Co., N. Y. 475 pages, 187 illustrations, 25 in colors, cloth, $5.00.

This is a systematic treatise of the highest order, brought out with the highest grade of skill in the mechanic preparation of the book. We venture to suggest, however, that the translation, though grammatic retains too many German idioms.

Progressive Medicine, edited by Hobart A. Hare, M. D., and Leighton F. Appleton, M. D., Vol. 14, No. 3, September, 1912. Published by Lea & Febiger, Philadelphia and New York. Quarterly, $6.00 per annum.

This issue deals with the Thorax, Dermatology and Syphilis, Obstetrics, Nervous System. The respective contributors are Edward P. Davis, Wm. Ewart, Wm. S. Gottheil and Wm. G. Spiller. The usual standard of excellence is maintained, indeed the present contributors have realized better than some have done in the past, that their function was to review medical literature rather than present personal views and experience.

---

Clinical Bacteriology and Haematology for Practitioners, by W. D'Este Emery, M. D., B. Sc., London, published by P. Blakiston's Son & Co., Philadelphia. 4th edition, 274 pages, 50 illustrations, some in colors, $2.00.

A very convenient plan is followed of stating for each disease, the apparatus and stains necessary and, after a detailed description of technic, is given a practical note on "interpretation of results." Vaccine and analogous methods of treatment are mentioned, whenever applicable.

---

Consumption in General Practice, by H. Hyslop Thomson, M. D., D. P. H., Medical Superintendent of Liverpool Sanatorium, Oxford University Press, 335 pages, illustrated, $5.50.

The disease is considered under three general headings, Diagnosis, Prognosis and Treatment. Under each, the various problems are systematically grouped in chapters and we are impressed with the fact that the author has omitted nothing new and neglected nothing old. Certain administrative differences in the sanatoria of England and the U. S. are of special interest, particularly as they denote no essential difference of professional opinion.

---

Muscle Spasm and Degeneration in Intrathoracic Inflammations, their importance as diagnostic aids and their influence in producing and altering the well established physical signs, also a consideration of their part in the causation of changes in the bony thorax. And Light Touch Palpation, the possibility and practicability of delimiting normal organs and diagnosticating diseased conditions within the chest and abdomen by very light touch. Francis Marion Pottenger, A. M., M. D., LL. D., Monrovia, Calif. Published by the C. V. Mosby Co., St. Louis. 105 pages, 16 illustrations, $2.00.

This is a typic Mosby book, unclassifiable, with no respect for longitude in the selection of an author, lacking a background of a row of conventional books on the same subject with which it may be compared. It represents three years of original observation on a large number of clinical cases, carefully controlled by a study of the work of other authors not along the same lines, but of such a nature as to afford data bearing upon the correctness of the author's conclusions. It puts new tools into the hand of the diagnostician and, obviously, he must learn to handle them before he can judge of their usefulness. Many probably can never learn to use them—any more than they have been able to use ausculatory percussion, for example.

---

THE SURGERY OF THE RECTUM FOR PRACTITIONERS, by Sir Frederick Wallis, M. B., B. C., F. R. C. S., London, published by the Oxford University Press, 355 pages, 129 illustrations, $5.50.

While the work is essentially surgical, full attention is given to clinical details and the work is not surgical in the narrow sense of dealing only with operations. Colitis and sigmoiditis and the extension of venereal diseases are also considered. It is rather a relief to note the absence of of X-ray plates.

---

PRACTICAL ANATOMY, from the Topographic Standpoint and a guide to dissection. John C. Heisler, M. D., Philadelphia. 366 illustrations, 225 in color by E. F. Faber. 790 pages including a thoroug hindex. Limp leather, $4.50. Published by J. B. Lippincott, Philadelphia.

From the fact that this book comes in a package bearing the advice that the medical student should get another anatomy, we infer that the present book is intended only as a dissecting and operating manual. The arrangement, too, is by regions instead of following the anatomic systems and apparatuses. Yet the text is full and, as under the description of certain articulations, even the physiology of movement is well discussed. In other words, while not intended to supplant the systematic works on anatomy, it is considerably more than a perfunctory regional treatise.

---

A MANUAL OF PHARMACY FOR PHYSICIANS, M. F. DeLorme, M. D., Ph. G., L. I. College Hospital, New York. 3d edition, cloth 221 pages, 19 illustrations, $1.25. P. Blakiston's Son & Co., Philadelphia.

This book follows the natural arrangement, discussing the general principles first and then the various official preparations by Galenical terms. We would suggest that there is no use writing a metric prescription which is merely thought out in apothecaries' terms and transposed. The table of Latin words used in prescriptions is complete and should obviate many of the mistakes made through ignorance or carelessness.

MEDICAL DIRECTORY OF NEW YORK, NEW JERSEY AND CONNECTICUT, 1912.

This book, compiled by the State Society, credited for the actual work being modestly withheld, appears in the customary form. The following comparative statistics may be interesting:

| | 1910 | 1911 | 1912 |
|---|---|---|---|
| Connecticut | 1384 | 1401 | 1394 |
| New Jersey | 2558 | 2582 | 2674 |
| New York State | 13474 | 13641 | 13696 |
| Greater New York City | 7169 | 7249 | 7365 |
| Up-state | 6305 | 6392 | 6331 |

These statistics are encouraging as indicating that we have reached a point at which the economic evil of professional over-supply will probably tend to be corrected by natural causes.

The JOURNAL will gladly serve as a bureau for revision of the Directory, for western New York. Glancing through the Buffalo list, we find the names of five physicians who moved away, a year or two ago. It should also be realized that the list—presumably for the entire scope of the directory—includes those licensed to practice. On the one hand, it omits the large number of irregular practitioners who may or may not be considered as competitors. On the whole, our personal experience is that such practitioners rather increase than decrease the work of the regular physician (using the word regular in a broad sense, for the directory is based on legal not ethical status). On the other hand, a considerable number of retired physicians and those engaged in teaching, clerical work, manufacturing, etc., are included. In 1911, the Buffalo list numbered 667, 22 of whom could be immedately excluded on account of death, non-residence, retirement, etc. The list of 1912 numbers 703, 27 of whom may be immediately excluded, besides several married women physicians not in active practice. 4 names should be added.

---

SULZER'S SHORT SPEECHES. J. B. Ogilvie Publishing Co., 57 Rose St., New York City. 25 cents, paper cover.

We can scarcely review this book without entering a field which a medical journal should carefully avoid but we may say that the versatility of a man in political life is interesting to a profession trained to avoid matters on which they can not work for years and that, irrespective of political views, the thoughts expressed are of interest to the average reader.

